# PlantCircNet: a database for plant circRNA–miRNA–mRNA regulatory networks

**DOI:** 10.1093/database/bax089

**Published:** 2017-12-09

**Authors:** Peijing Zhang, Xianwen Meng, Hongjun Chen, Yongjing Liu, Jitong Xue, Yincong Zhou, Ming Chen

**Affiliations:** Department of Bioinformatics, The State Key Laboratory of Plant Physiology and Biochemistry, Institute of Plant Science, College of Life Sciences, Zhejiang University, Hangzhou 310058, China; James D. Watson Institute of Genome Sciences, Zhejiang University, Hangzhou 310058, China

## Abstract

Circular RNA (circRNA) is a novel type of endogenous noncoding RNA with covalently closed loop structures, which are widely expressed in various tissues and have functional implications in cellular processes. Acting as competing endogenous RNAs (ceRNAs), circRNAs are important regulators of miRNA activities. The identification of these circRNAs underlines the increasing complexity of ncRNA-mediated regulatory networks. However, more biological evidence is required to infer direct circRNA–miRNA associations while little attention has been paid to circRNAs in plants as compared to the abundant research in mammals. PlantCircNet is presented as an integrated database that provides visualized plant circRNA–miRNA–mRNA regulatory networks containing identified circRNAs in eight model plants. The bioinformatics integration of data from multiple sources reveals circRNA–miRNA–mRNA regulatory networks and helps identify mechanisms underlying metabolic effects of circRNAs. An enrichment analysis tool was implemented to detect significantly overrepresented Gene Ontology categories of miRNA targets. The genomic annotations, sequences and isoforms of circRNAs were also investigated. PlantCircNet provides a user-friendly interface for querying detailed information of specific plant circRNAs. The database may serve as a resource to facilitate plant circRNA research. Several circRNAs were identified to play potential regulatory roles in flower development and response to environmental stress from regulatory networks related with miR156a and AT5G59720, respectively. This present research indicated that circRNAs could be involved in diverse biological processes.

**Database URL**: http://bis.zju.edu.cn/plantcircnet/index.php

## Introduction

Circular RNA (circRNA) is a new class of endogenous noncoding RNA, which was dismissed as molecular flukes or by-products of aberrant splicing for nearly 20 years (1–4). The high-throughput transcriptome sequencing technology and bioinformatics approaches have enabled systematic discovery of circRNAs in various species (5–7). Different from linear mRNAs, circRNAs form covalently closed loop structures with neither 5′–3′ ends nor poly(A) tails, enabling them to escape from being degraded by RNase R (8, 9). Emerging evidence suggests that the majority of circRNAs are abundant, stable and conserved across species, are widespread in eukaryotic transcriptomes (10), and play important roles in diverse biological processes (9, 11). They were reported as competing endogenous RNAs or miRNA sponges (12, 13), and moreover they can regulate alternative splicing or transcription (14, 15). In addition, some evidence revealed that they have the potential to become promising biomarkers for disease diagnosis and prognosis (16, 17).

Evidence suggested that circRNAs regulate transcription and pathways by manipulating miRNAs. CircRNAs can function as miRNA sponges, which naturally sequester and competitively inhibit the activity of miRNAs (7, 12). CircRNAs and mRNAs with common miRNA target sites compete for miRNA binding and form a complex interactive and regulatory network, known as the ceRNA network (18). CiRS-7, an identified circRNA acting as a ceRNA or sponge of miR-7, competitively inhibits miR-7 activity and promotes oncogene expression (such as EGFR and XIAP), while it inhibits tumor suppressor genes (such as KLF4) and therefore promotes the initiation and development of cancer (13, 19).

The existing circRNA databases like circBase (20), starbase (21) and circNet (22), pay more attention to the animal circRNAs, such as those of *Homo sapiens*, *Caenorhabditis elegants* and *Mus musculus*. There have been fewer comprehensive studies on plant circRNAs than those in animals. Given the emerging research efforts of plant circRNAs and their biological importance, we constructed a database called PlantCircNet to decipher circRNA regulatory roles and store related resources. Previously reported and newly identified plant circRNAs from eight model species are cataloged, of which detailed information can be queried and downloaded from the database, including genomic loci, parental gene, unique reads and isoform sequences. For each entry, expression level and genome browser are also presented in detail. Embracing the idea that circRNAs are enriched with conserved miRNA binding sites and function as natural miRNA sponges, PlantCircNet maps circRNA–miRNA–mRNA interactions into visualized regulatory networks. The networks can be of importance in interpreting the functions of circRNAs and identifying circRNA-related mechanisms. For example, the miR156a-related subnetwork in PlantCircNet indicates that the circRNAs may be involved in diverse biological processes, especially flower development, response to stress and photoperiodism and the AT5G59720-subnetwork interprets the potential significance of the putative regulatory networks. The associated network, circRNA names and sequences are accessible in PlantCircNet by searching an interested gene or miRNA. Furthermore, an enrichment analysis tool was implemented to detect significantly overrepresented Gene Ontology (GO) categories of miRNA targets.

Above all, PlantCircNet provides a user-friendly interface for circRNA–miRNA–mRNA regulatory networks, including database search, information browsing and interaction visualization.

## Materials and methods

**Figure 1.**
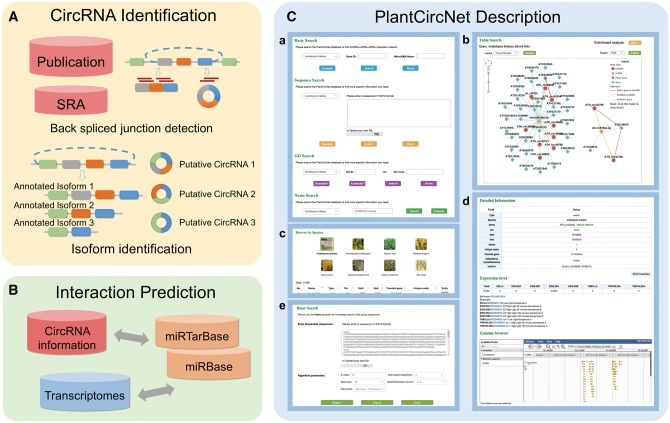
Overview of PlantCircNet workflow. (**A**) Identification of plant circRNAs. (**B**) Prediction of circRNA–miRNA–mRNA interactions. (**C**) Features of PlantCircNet, (**Ca**) search page, (**Cb**) network visualization, (**Cc**) browse page, (**Cd**) detail page and (**Ce**) blast search.

The workflow of PlantCircNet is summarized in Figure 1, including circRNA identification, circRNA–miRNA–mRNA interactions prediction and PlantCircNet description.

### Data collection

Reported plant back-spliced junction sites were manually collected from the Supplementary Information provided from existing high confidence studies (23–27). In addition, 119 publicly available RNA-seq samples (Supplementary Table S1) were collected from a wide range of independent experiments across 8 species from the NCBI Sequence Read Archive (28). CircRNA back-spliced junction sites were identified within these samples. A pipeline was designed for acquiring the expression patterns of circRNAs within these samples.

All reference genomes, transcriptomes and annotation files were downloaded from Ensembl (http://www.ensembl.org/index.html), and TAIR10 (http://www.arabidopsis.org/) for *Arabidopsis thaliana* (Supplementary Table S2). MiRNA information of eight plant species were retrieved from miRBase (http://www.mirbase.org/) (29). For GO enrichment analysis, the ontology was downloaded from GO Consortium (http://geneontology.org/), and GO annotation files were downloaded from agriGO (http://bioinfo.cau.edu.cn/agriGO/index.php) (30).

### CircRNA identification

The key point for plant circRNA identification is to find back-spliced junction sites. The algorithms of several tools for circRNA identification were compared and evaluated (31). A predictive model was built to identify novel circRNAs, which is a combination of tools including find_circ (7), CIRI (32), MapSplice (33), CircRNAFinder (34) and UROBORUS (35). First, we used the five tools to collect all putative back-spliced junction sites. Then a pseudoRef file was created with the flanking sequences of those sites. Third, raw reads were mapped to the pseudoRef file, which confirmed putative back-spliced junction sites with the false positive sites filtered out. CircRNAs with small misalignments (two bases) for the back-spliced junction sites were considered the same. Moreover, candidate circRNAs must be supported by at least two unique back spliced reads.

To obtain the full length nucleotide sequence of candidate circRNAs, circRNA isoforms possibly originating from the same back-spliced junction site need to be considered (6, 36). These isoforms were identified with annotated transcripts based on the method proposed by Salzman *et al.* (6) These circRNAs were classified into ‘exonic’, ‘intronic’, ‘UTR’, ‘intergenic’ and ‘other’ based on how their starts and ends are aligned with genes. ‘Other’ stands for the circRNAs whose back spliced reads were aligned to two or more different genes.

### CircRNA–miRNA–mRNA interactions

TargetFinder, which was specifically developed for the identification of plant small RNA targets, was applied to predict the miRNA-target interactions (37). The score parameter is set as 3 to improve the reliability of the predicted results, as suggested in PceRBase (38). Candidate circRNA sequences of each plant species were used as the source for the prediction of circRNA–miRNA interactions. The source for the prediction of miRNA–mRNA interactions are the whole transcriptomes of each plant species, and the predicted target genes are further incorporated with experimentally validated datasets from miRTarBase (39).

## Utility

### Database implementation

Currently, PlantCircNet operates on a Linux, Apache, MySQL and PHP stack. The web server is running with online BLAST program for sequence blast search, Cytoscape web program (40) for visualization of circRNA-associated networks and JBrowse (41) as the genome browser section.

### Query option

Four search options are provided: (i) Basic Search, (ii) Sequence Search, (iii) GO Search and (iv) Name Search (Figure 1Ca) for the database accessibility.

The basic search option provides an interface for querying the putative circRNA-associated regulatory network with a selected species, a gene ID and a miRNA name. A circRNA–miRNA–mRNA network associating with the queried term will be shown in the result page.

The sequence search requires circRNA sequences in fasta format, either as text or an uploaded file. The species should be selected before submission. Users can get a list of queried circRNA–miRNA interactions and potential circRNA-associated network with their own sequences.

The GO search option allows the user to search for circRNAs by a specific GO id or GO term. Users can get a list of putative related circRNAs according to the queried gene function.

The name search option is for quickly matching the circRNA with a specific name.

### Network visualization

The post-transcriptional regulatory relationships of (i) circRNAs originating from the given gene and (ii) miRNAs targeting the given gene should be given in the search result. In the network visualization, nodes (genes, miRNAs and circRNAs) and edges (interactions) are differently colored according to their types, and their positions can be displayed in three different layouts. The size of nodes and the distance between nodes do not indicate any biological meaning, but are automatically generated according to a chosen layout. The interactive network allows users to check the known interaction information for each circRNA/miRNA/mRNA by clicking on the node. Besides, the details of all circRNAs appearing in the network will be shown below the network diagram, containing genomic loci, type, parental gene and unique reads. ‘GO Enrichment Analysis’ module has been developed for the functional enrichment analysis of miRNA target genes in the network. Hopefully, this information will effectively aid users for exploring the regulatory roles of circRNA. Images of networks could be exported in several formats if necessary.

### CircRNA information

All the circRNA information of eight plant species are also available in PlantCircNet. Downloadable circRNA data of selected plant species were listed in the web server. The Details page could be divided in three sections. The Detailed information section contains the type, unique reads, parental gene and isoforms of the circRNA. All the interaction relationships of this circRNA will be shown in a network, which contains the circRNA, its parental gene, related miRNAs and the possibly existing target genes of these miRNAs. The Expression level section contains the expression profiles of each circRNA across several samples, estimated through RPM (Reads Per Million fragments mapped) values. The Genome browser section contains the genomic sequences and all circRNAs as well as overlapped linear transcripts around the selected area.

## Results

### Database contents

PlantCircNet aims to provide circRNA–miRNA–mRNA interaction networks specifically for plants. Currently, the database covers eight model species, containing 139 276 circRNAs, 96 418 of which were obtained from previously reported works. Significantly, most of the circRNAs are so-called exonic circRNAs which are generated from exons of a single protein-coding gene (Table 1). In total, 16,020 CircRNA–miRNA interactions and 27,872 miRNA–mRNA interactions are also contained, from the predicted results and experimentally validated data sets. Furthermore, the reliability of some circRNAs identified by software prediction was confirmed by comparison with experimentally validated circRNAs. Twenty-six of 27 circRNAs in rice and 47 of 61 in barley were consistent with experimental data (Supplementary Table S3). This result suggested the accuracy of our prediction.

**Table 1.** Statistics of circRNAs in PlantCircNet

	Arabidopsis thaliana	Brachypodium distachyon	Glycine max	Hordeum vulgare	Oryza sativa	Solanum lycopersicum	Triticum aestivum	Zea mays
Exonic	90 521 (94.2%)	352 (43.7%)	598 (48.9%)	1928 (44.4%)	14 672 (58.1%)	3513 (55.2%)	1758 (45.6%)	828 (63.4%)
Intronic	53 (0.0%)	15 (1.9%)	17 (1.4%)	99 (2.3%)	795 (3.1%)	92 (1.4%)	18 (0.5%)	13 (1.0%)
UTR	0 (0.0%)	0 (0.0%)	0 (0.0%)	0 (0.0%)	421 (1.7%)	0 (0.0%)	0 (0.0%)	0 (0.0%)
Intergenic	87 (0.1%)	32 (4.0%)	17 (1.4%)	338 (7.8%)	2221 (8.8%)	374 (5.9%)	2070 (53.7%)	71 (5.4%)
Other	5474 (5.7%)	406 (50.4%)	590 (48.3%)	1977 (45.5%)	7140 (28.3%)	2384 (37.5%)	7 (0.2%)	395 (30.2%)
Total	96 135	805	1222	4342	25249	6363	3853	1307

### MiR156a-related circRNA–miRNA–mRNA network in Arabidopsis

**Figure 2.**
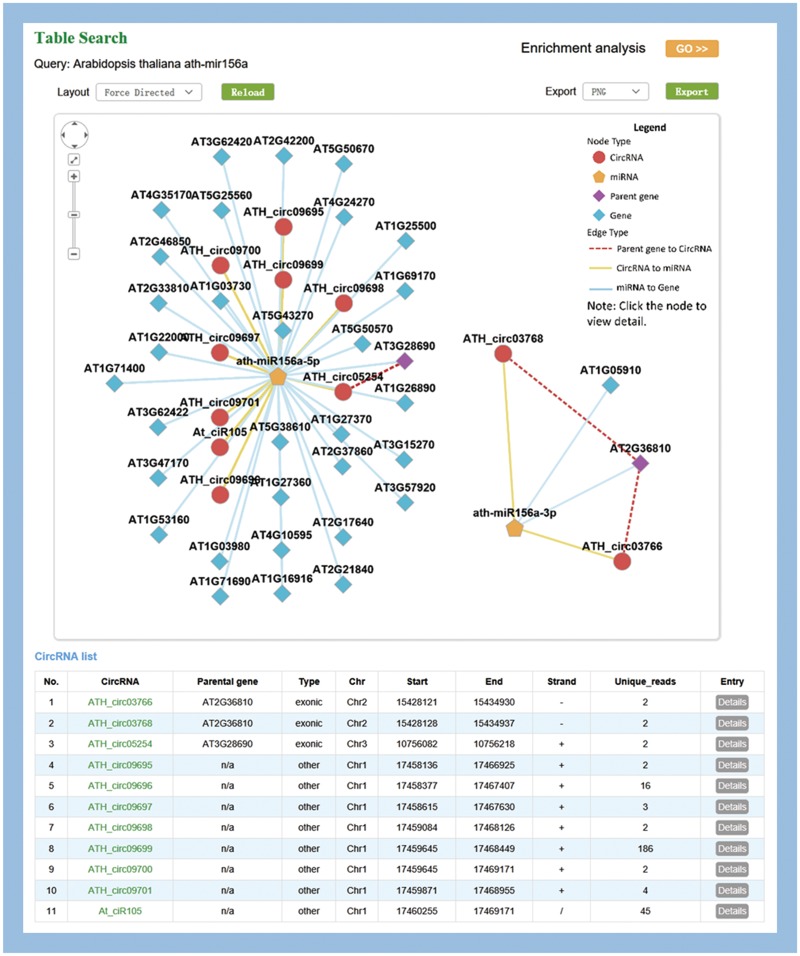
Visualization of miR156a-related circRNA–miRNA–mRNA network. The output contains two independent subnetworks of the miRNAs, ath-miRNA156a-5p and ath-miRNA156a-3p, by a fuzzy search.

MiR156 is one of the most abundant and evolutionarily conserved miRNAs in plants (42). As an example, ath-miR156a was used to illustrate the information provided by PlantCircNet. After ath-miR156a was entered as a key word in *A. thaliana*, PlantCircNet provided an integrated circRNA–miRNA–mRNA network (Figure 2), including circRNAs and mRNAs that are putatively targeted by ath-miR156a-5p, together with the relationships of circRNAs and their parental genes. Some of the targets have been validated, such as AT1G27370, AT1G53160, AT3G15270 and AT5G43270 (43). GO enrichment analysis (Figure 2) for the genes in the ath-miR156a-related network shows that they are involved in diverse biological processes, especially flower development, response to stress and photoperiodism (*P*-value < 0.01, Supplementary Table S4).

Arabidopsis miR156 is known to play important roles in temperature responses and regulating flowering time (44), which is consistent with the result. As illustrated in Supplementary Table S5, circRNAs in the network showed differential expression in diverse stress, especially high light intensity. Together, these results indicate that the circRNAs involved in this network may play important roles in these processes.

### AT5G59720-subnetwork as an example to interpret the potential significance of the putative regulatory networks

**Figure 3.**
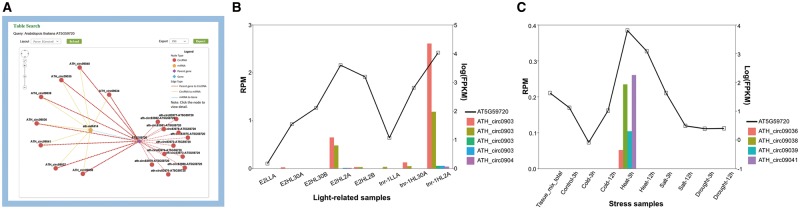
AT5G59720-subnetwork as an example to interpret the potential significance of the putative regulatory networks. (**A**) Screenshots of circRNAs–miR414–AT5G59720 regulatory network. (**B**) Expression level of AT5G59720 and related circRNAs among different light treatments. Samples (from left to right) repre-sent for E2 (ELIP2 promoter) low light A, E2 high-light 30 min A, E2 high-light 30 min B, E2 high-light 120 min A, E2 high-light 120 min B, tnr-1 (tanorexia-1) low light A, tnr-1 high-light 30 min A and tnr-1 high-light 120 min A. (**C**) Expression level of AT5G59720 and related circRNAs among different stress treatments. Samples (from left to right) represent for mix tissue, control 3 h, cold 3 h, cold 12 h, heat 3 h, heat 12 h, salt 3 h, salt 12 h, drought 3 h, drought 12 h.

The miR156a example suggested miRNAs’ inspiring role for circRNA studies, while genes can also be used to infer the functions of circRNAs. To further illustrate how the network might help researchers, we presented another example in which a gene was used as the input for a query. The gene AT5G59720 (Heat Shock Protein 18.2, HSP18.2) encodes a low-molecular-weight heat shock protein that contains the heat shock element in the promoter region (45). The expression is up-regulated in response to heat shock, as well as high-light stress (46, 47). After AT5G59720 was queried in *A. thaliana*, a circRNA–miRNA–mRNA subnetwork was provided (Figure 3A). This network showed 19 circRNAs generated from AT5G59720, all of which could be targeted by ath-miR414, the only miRNA in this network. Among these circRNAs, 8 were detected by our predictive model, whereas 11 were obtained from previously reported works. As a conserved miRNA in plant, miR414 plays important roles in different processes during plant growth and development, especially in responding to changes in specific environmental conditions, including temperature, light and oxygen (48, 49), and was identified as a putative regulator of AT5G59720 (50). Further, the circRNAs detected by the predictive model were found to be specifically expressed in light-related (PRJNA218215) and stress-related (PRJNA213635) samples. Particularly, ATH_circ09039 was correlated to both types. Transcript-level expression analysis showed that AT5G59720 positively correlated with levels of those eight circRNAs, which is up-regulated in high light time (Figure 3B) and high temperature condition (Figure 3C). These findings imply that these eight circRNAs may function as ceRNAs in the miR414-involved regulatory process of AT5G59720 expression level, by sponging miR414. The results demonstrated the significance of the network provided in our database.

## Discussion

Recent research exposed diverse functions of circRNAs, especially as competing endogenous RNAs or miRNA sponges. Therefore, circRNA-related regulatory networks are considered quite useful for discovering the specific functions of circRNAs and the processes they are involved in. In this article, we have presented PlantCircNet, a comprehensive database of plant circRNAs-associated regulatory networks. It also provides browse and search functions for circRNA information.

Many circRNA databases have emerged recently, but few databases focused on plants. It is believed that PlantCircNet serves as the most comprehensive resource of plant circRNAs and exceeds those databases with good response time. Compared with PlantcircBase (51), another plant circular RNA dababase, PlantCircNet provides a larger collection of plant circRNAs and its miRNA-related interactions, with more plant species and circRNA isoforms. Besides, PlantCircNet allows users to apply multiple circRNA search options and provides visualized circRNA–miRNA–mRNA regulatory networks.

In summary, PlantCircNet provides a unique data resource for plant circRNAs and putative circRNA-associated regulatory networks. These results will help researchers to detect the potential circRNAs quickly, and provide guidance on studying circRNA functions and related pathways. To include new resources and strengthen the predictive value of PlantCircNet platform, the database will be updated annually, with the addition of more available plant species and experimentally validated plant circRNAs.

## Supplementary data

Supplementary data are available at *Database* Online.

## Supplementary Material

Supplementary Data
