# Supplementary material for: PlantCircNet: a database for plant circRNA–miRNA–mRNA regulatory networks
Source: Database (Oxford). 2017 Dec 9;2017:bax089. doi: 10.1093/database/bax089 (PMC5727401; doi:10.1093/database/bax089)
Supplement: Supplementary Data [file bax089_supp.pdf]

Supplementary Information Tables S1–S5

Table S1. The publicly available RNA-sequencing datasets used in this study

Species	Length of reads	NCBI Accession	Samples ID
Arabidopsis thaliana	101bp	PRJNA218215	SRR1004790, SRR1004791, SRR1004829, SRR1004830, SRR1004831, SRR1004832, SRR1004833, SRR1004834
	100bp	PRJNA213635	SRR944362, SRR944363, SRR944364, SRR944365, SRR944366, SRR944367, SRR944368, SRR944369, SRR944370, SRR944371
Brachypodium distachyon	50bp	PRJNA273411	SRR1772223, SRR1772224, SRR1772225, SRR1772226, SRR1772227, SRR1772228, SRR1772229, SRR1772230, SRR1772231, SRR1772232, SRR1772233, SRR1772234, SRR1772235, SRR1772236, SRR1772237, SRR1772238, SRR1850587, SRR1850670, SRR1850671, SRR1850716
Glycine max	50bp	PRJNA285677	SRR2048167, SRR2048168, SRR2048169, SRR2048170, SRR2048171, SRR2048172, SRR2048173, SRR2048174, SRR2048175, SRR2048176, SRR2048177, SRR2048178, SRR2048179, SRR2048180, SRR2048181, SRR2048182, SRR2048183, SRR2048184, SRR2048185, SRR2048186, SRR2048187, SRR2048188, SRR2048189, SRR2048190, SRR2048191, SRR2048192, SRR2048193, SRR2048194, SRR2048195, SRR2048196, SRR2048197, SRR2048198, SRR2048199, SRR2048200, SRR2048201, SRR2048202
Hordeum vulgare	101bp	PRJNA295482	SRR2379177, SRR2379209, SRR2379274, SRR2379393, SRR2379445, SRR2379446, SRR2379577, SRR2379602, SRR2379664, SRR2379887, SRR2379950, SRR2379972, SRR2380048, SRR2380049, SRR2380116
Oryza sativa	101bp	PRJNA215013	SRR1005257, SRR1005258, SRR1005259, SRR1005260, SRR1005261, SRR1005262, SRR1005302, SRR1005303, SRR1005304, SRR1005305, SRR1005306, SRR1005307, SRR1005320, SRR1005321, SRR1005322, SRR1005323, SRR1005324, SRR1005325, SRR1005365, SRR1005366, SRR1005367, SRR1005368, SRR1005369, SRR1005370
Solanum lycopersicum	100bp	PRJNA255271	SRR1514810, SRR1609960, SRR1609961, SRR2102073
Zea mays	100bp	PRJNA287309	SRR2070626, SRR2070627

Table S2. The reference genomes, transcriptomes and annotation files used in this study

Species	Species ID	Common Name	Release Version	Source
Arabidopsis thaliana	ath	Arabidopsis	TAIR10	http://www.arabidopsis.org/
Brachypodium distachyon	bdi	stiff brome	v1.0	ftp://ftp.ensemblgenomes.org/pub/current/plants/
Glycine max	gma	soybean	V1.0	ftp://ftp.ensemblgenomes.org/pub/current/plants/
Hordeum vulgare	hvu	barley	ASM32608v1	ftp://ftp.ensemblgenomes.org/pub/current/plants/
Oryza sativa	osa	rice	IRGSP-1.0	ftp://ftp.ensemblgenomes.org/pub/current/plants/
Solanum lycopersicum	sly	tomato	SL2.50	ftp://ftp.ensemblgenomes.org/pub/current/plants/
Zea mays	zma	maize	AGPv3	ftp://ftp.ensemblgenomes.org/pub/current/plants/

Table S3. Predicted circRNAs consistent with experimentally validated RNAs by comparison of back-spliced junction sites

Species	Experimentally identified circRNAs	Chr	Start	End	circRNA	Reference
Oryza sativa	Chr1_22121499_22122046	Chr1	22121499	22122046	OSA_circ00869	PMID: 27739910
Oryza sativa	Chr11_13707993_13708205	Chr11	13707993	13708205	OSA_circ08079	PMID: 27739910
Oryza sativa	Chr10_20890579_20890904	Chr10	20890579	20890904	OSA_circ07792	PMID: 27739910
Oryza sativa	Chr10_23115090_23115651	Chr10	23115090	23115651	OSA_circ07893	PMID: 27739911
Oryza sativa	Chr11_21179446_21180854	Chr11	21179446	21180854	OSA_circ08151	PMID: 27739912
Oryza sativa	Chr12_3795288_3795604	Chr12	3795288	3795604	OSA_circ08363	PMID: 27739913
Oryza sativa	Chr3_11550444_11551137	Chr3	11550444	11551137	OSA_circ03522	PMID: 27739914
Oryza sativa	Chr4_29982442_29982821	Chr4	29982442	29982821	OSA_circ04545	PMID: 27739915
Oryza sativa	Chr5_17342446_17342852	Chr5	17342446	17342852	OSA_circ05013	PMID: 27739916
Oryza sativa	Chr5_28580407_28580547	Chr5	28580407	28580547	OSA_circ05293	PMID: 27739917
Oryza sativa	Chr5_29522019_29522678	Chr5	29522019	29522678	OSA_circ05328	PMID: 27739918
Oryza sativa	Chr5_3617186_3617467	Chr5	3617186	3617467	OSA_circ04874	PMID: 27739919
Oryza sativa	Chr7_11593140_11593419	Chr7	11593140	11593419	OSA_circ06066	PMID: 27739920
Oryza sativa	Chr7_28426089_28426740	Chr7	28426089	28426740	OSA_circ06338	PMID: 27739921
Oryza sativa	Chr8_19735841_19736366	Chr8	19735841	19736366		PMID: 27739922
Oryza sativa	Chr3_267627_268062	Chr3	267627	268062	OSA_circ09450	PMID: 27739923
Oryza sativa	Chr4_35476588_35477345	Chr4	35476588	35477345	OSA_circ04754	PMID: 27739924
Oryza sativa	Os_ciR133	Chr3	11550444	11551137	OSA_circ03522	PMID: 26204923
Oryza sativa	Os_ciR182	Chr5	29522019	29522678	OSA_circ05328	PMID: 26204923
Oryza sativa	Os_ciR190	Chr5	3617186	3617467	OSA_circ04874	PMID: 26204923
Oryza sativa	Os_ciR158	Chr1	21424261	21426198	OSA_circ00838	PMID: 26204923
Oryza sativa	Os_ciR308	Chr11	21179446	21180854	OSA_circ08150	PMID: 26204923
Oryza sativa	Os_ciR455	Chr5	28580407	28580547	OSA_circ05293	PMID: 26204923
Oryza sativa	Os_ciR207	Chr3	27106436	27107773	OSA_circ09523	PMID: 26204923
Oryza sativa	Os_ciR239	Chr3	30220074	30221928	OSA_circ11640	PMID: 26204923
Oryza sativa	Os_ciR198	Chr7	28426089	28426740	OSA_circ06338	PMID: 26204923
Oryza sativa	Os_ciR300	Chr5	17342446	17342852	OSA_circ05013	PMID: 26204923
Hordeum vulgare	Cax2_circular RNA	Chr4	356281355	356282249	HVU_circ03367	PMID: 27375638

Hordeum vulgare	Ctr1 like_circular RNA	Chr3	351423944	351425007	HVU_circ03016	PMID: 27375639
Hordeum vulgare	MicroRNA1126_circular RNA	Chr2	467982888	467983386	HVU_circ02772	PMID: 27375640
Hordeum vulgare	Aux1_circular RNA	Chr3	486789367	486789848	HVU_circ00990	PMID: 27375641
Hordeum vulgare	AbcI3_circular RNA	Morex_contig1661226	956	1173		PMID: 27375642
Hordeum vulgare	18S rRNA_circular RNA1	Chr1	429515268	429515439	HVU_circ02592	PMID: 27375643
Hordeum vulgare	18S rRNA_circular RNA2	Chr1	429515283	429515435	HVU_circ02596	PMID: 27375644
Hordeum vulgare	18S rRNA_circular RNA3	Chr1	429515253	429515394	HVU_circ02588	PMID: 27375645
Hordeum vulgare	18S rRNA_circular RNA4	Chr1	429515283	429515394	HVU_circ02595	PMID: 27375646
Hordeum vulgare	18S rRNA_circular RNA5	Chr1	429515275	429515370	HVU_circ02104	PMID: 27375647
Hordeum vulgare	18S rRNA_circular RNA6	Chr1	429515253	429515403	HVU_circ02589	PMID: 27375648
Hordeum vulgare	18S rRNA_circular RNA7	Chr1	429513196	429513343		PMID: 27375649
Hordeum vulgare	Internal transcribed spacer 2 of 18S,5.8S,26S rRNA_circular RNA1	Chr1	429512475	429512569	HVU_circ02578	PMID: 27375650
Hordeum vulgare	Internal transcribed spacer 2 of 18S,5.8S,26S rRNA_circular RNA2	Chr1	177434203	177434347		PMID: 27375651
Hordeum vulgare	Nad9_circular RNA	Morex_contig_70567	2809	3204		PMID: 27375652
Hordeum vulgare	Mit. Apocytochrome b_circular RNA	Morex_contig_42365	12844	13477		PMID: 27375653
Hordeum vulgare	Cox1_circular RNA1	Chr1	23865393	238657407		PMID: 27375654
Hordeum vulgare	Cox1_circular RNA2	Chr1	23865228	23865597	HVU_circ02390	PMID: 27375655
Hordeum vulgare	Cox1_circular RNA3	Chr1	23865348	23865740	HVU_circ00286	PMID: 27375656
Hordeum vulgare	Cox1_circular RNA4	Chr1	23864364	23866057	HVU_circ00336	PMID: 27375657
Hordeum vulgare	Cox1_circular RNA5	Chr1	23864474	23866037	HVU_circ00155	PMID: 27375658
Hordeum vulgare	Cox1_circular RNA6	Chr1	23864318	23866037	HVU_circ00037	PMID: 27375659
Hordeum vulgare	Cystathionine beta lyase_circular RNA	Chr7	53946083	53946582	HVU_circ01734	PMID: 27375660
Hordeum vulgare	BZip11_circular RNA	Chr6	52088926	52089401	HVU_circ03830	PMID: 27375661
Hordeum vulgare	ADP ribosylation factor 1_circular RNA	Chr1	389129363	389130325	HVU_circ02532	PMID: 27375662
Hordeum vulgare	Sec23/Sec24 transport protein_circular RNA	Chr2	482080734	482081397	HVU_circ00698	PMID: 27375663
Hordeum vulgare	Fumarase 2 _circular RNA	Chr4	243159573	243159671		PMID: 27375664
Hordeum vulgare	Inositol transporter 2_circular RNA	Chr2	483514445	483514888	HVU_circ00699	PMID: 27375665

Hordeum vulgare	Formin like protein 20_circular RNA1	Chr4	491530198	491530553		PMID: 27375666
Hordeum vulgare	Formin like protein 20_circular RNA1	Chr4	491530332	491530553		PMID: 27375667
Hordeum vulgare	Alpha mannosidase 1_circular RNA	Chr2	566491430	566492027	HVU_circ00798	PMID: 27375668
Hordeum vulgare	Chromosome segregation protein_circular RNA	Chr5	361207403	361207861		PMID: 27375669
Hordeum vulgare	Laccase 12_circular RNA	Chr3	499962355	499962651	HVU_circ03115	PMID: 27375670
Hordeum vulgare	ARID/BRIGHT DNA binding protein_circular RNA	Chr2	532048153	532048410	HVU_circ02138	PMID: 27375671
Hordeum vulgare	Ubiquitin conjugating enzyme 11_circular RNA	Chr3	473869516	473869731	HVU_circ03099	PMID: 27375672
Hordeum vulgare	Probable microtubule stabilizing protein_circular RNA	Chr4	330511991	330512323		PMID: 27375673
Hordeum vulgare	RNA binding (RRM/RBD/RNP motifs protein_circular RNA	Chr4	252670767	252671078	HVU_circ01152	PMID: 27375674
Hordeum vulgare	RNA binding (RRM/RBD/RNP motifs protein_circular RNA	Chr6	308455217	308455370	HVU_circ01625	PMID: 27375675
Hordeum vulgare	RNA binding (RRM/RBD/RNP motifs protein_circular RNA	Chr6	260427394	260427518	HVU_circ01604	PMID: 27375676
Hordeum vulgare	Ribosomal protein L30/L7_circular RNA	Chr5	524054971	524055497	HVU_circ01625	PMID: 27375677
Hordeum vulgare	Transducin/WD40 repeat like protein_circular RNA	Chr5	484562574	484563329	HVU_circ01416	PMID: 27375678
Hordeum vulgare	Abscisic acid responsive (TB2/DP1, HVA22 protein_circular RNA	Chr6	20988426	20990992	HVU_circ01496	PMID: 27375679
Hordeum vulgare	Far upstream element binding protein 2 _circular RNA	Chr6	245908278	245908734	HVU_circ03867	PMID: 27375680
Hordeum vulgare	Sec independent protein translocase _circular RNA	Morex_contig_10645[illegible]	2137	3175		PMID: 27375681
Hordeum vulgare	Ubiquitin specific protease 17_circular RNA	Chr7	307483097	307485269	HVU_circ04178	PMID: 27375682

Hordeum vulgare	Probable KH domain containing splicing factor_circular RNA	Chr7	457516955	457517152	HVU_circ04202	PMID: 27375683
Hordeum vulgare	Ribosomal protein L6_circular RNA	Chr6	245896672	245897042	HVU_circ01588	PMID: 27375684
Hordeum vulgare	Glycyl tRNA synthetase 2_circular RNA2	Chr6	268555508	268556210	HVU_circ01610	PMID: 27375685
Hordeum vulgare	Glycyl tRNA synthetase 2_circular RNA2	Chr6	268555508	268556362	HVU_circ01611	PMID: 27375686
Hordeum vulgare	Kinesin related protein 11 like_circular RNA	Chr1	128008822	128014221	HVU_circ00397	PMID: 27375687
Hordeum vulgare	SAD1/UNC 84 domain protein 2_circular RNA	Chr1	60689841	60690191	HVU_circ00356	PMID: 27375688
Hordeum vulgare	Probable beta-1-4-glucosyltransferase_circular RNA	Chr4	71095325	71097097	HVU_circ01081	PMID: 27375689
Hordeum vulgare	Unknown_circular RNA	Chr7	4237959	4238849		PMID: 27375690
Hordeum vulgare	Unknown_circular RNA	Chr2	476367671	476368169	HVU_circ02134	PMID: 27375691
Hordeum vulgare	Probable long non coding RNA_circular RNA	Chr2	605441109	605441443	HVU_circ02152	PMID: 27375692
Hordeum vulgare	Unknown_circular RNA	Chr3	100501939	100502294	HVU_circ02165	PMID: 27375693
Hordeum vulgare	Probable long non coding RNA_circular RNA	Chr4	247058951	247059280	HVU_circ03322	PMID: 27375694
Hordeum vulgare	Probable aminopeptidase_circular RNA	Chr5	286201637	286202872	HVU_circ03589	PMID: 27375695
Hordeum vulgare	Unknown_circular RNA	Chr5	6888869	6889034	HVU_circ03486	PMID: 27375696
Hordeum vulgare	Probable long non coding RNA_circular RNA	Chr5	53926495	53926935		PMID: 27375697
Hordeum vulgare	Probable long non coding RNA_circular RNA	Chr1	363958561	363959513	HVU_circ02523	PMID: 27375697

Table S4. Gene ontology (GO) enrichment analysis for the genes in the miR156a-related network

Biological Process			
**GO Term**	**Description**	**Genes**	**P-value**
GO:0000103	sulfate assimilation	[AT2G17640]	1.88E-02
GO:0000302	response to reactive oxygen species	[AT2G37860]	3.24E-02
GO:0006355	regulation of transcription, DNA-templated	[AT2G42200] [AT1G27360] [AT1G53160] [AT3G57920] [AT1G69170] [AT5G50570] [AT3G15270] [AT5G43270]	5.36E-04
GO:0006535	cysteine biosynthetic process from serine	[AT2G17640]	1.88E-02
GO:0009267	cellular response to starvation	[AT3G62420]	8.83E-03
GO:0009648	photoperiodism	[AT2G37860]	7.57E-03
GO:0009827	plant-type cell wall modification	[AT2G33810] [AT3G57920]	2.00E-02
GO:0009860	pollen tube growth	[AT2G33810] [AT3G57920]	3.79E-02
GO:0009886	post-embryonic animal morphogenesis	[AT2G33810]	7.57E-03
GO:0009911	positive regulation of flower development	[AT2G33810]	4.70E-02
GO:0010038	response to metal ion	[AT1G03980]	1.01E-02
GO:0010228	vegetative to reproductive phase transition of meristem	[AT2G42200] [AT2G33810] [AT3G57920]	5.79E-03
GO:0010229	inflorescence development	[AT2G33810]	2.38E-02
GO:0010321	regulation of vegetative phase change	[AT1G53160] [AT2G33810] [AT3G15270]	6.50E-08
GO:0010358	leaf shaping	[AT1G27370]	1.01E-02
GO:0046938	phytochelatin biosynthetic process	[AT1G03980]	3.79E-03
GO:0048366	leaf development	[AT2G42200] [AT2G37860]	7.84E-03
GO:0048507	meristem development	[AT2G33810]	3.49E-02
GO:0048510	regulation of timing of transition from vegetative to reproductive phase	[AT1G27360] [AT1G27370] [AT5G43270]	9.02E-06
GO:0048653	anther development	[AT2G42200] [AT3G57920] [AT5G50570] [AT5G50670] [AT5G43270]	1.76E-08
GO:2000025	regulation of leaf formation	[AT2G42200]	3.79E-03
GO:2000693	positive regulation of seed maturation	[AT3G62420]	5.06E-03

Molecular Function

GO Term	Description	Genes	P-value
GO:0003700	transcription factor activity, sequence-specific DNA binding	[AT2G42200] [AT1G27360] [AT1G53160] [AT2G33810] [AT3G57920] [AT1G69170] [AT5G50570] [AT3G15270] [AT1G27370] [AT3G62420] [AT5G43270]	4.85E-06
GO:0009001	serine O-acetyltransferase activity	[AT2G17640]	7.57E-03
GO:0016756	glutathione gamma-glutamylcysteinyltransferase activity	[AT1G03980]	7.57E-03

Cellular Component

GO Term	Description	Genes	P-value
GO:0005634	nucleus	[AT2G42200] [AT2G36810] [AT1G27360] [AT1G26890] [AT1G05910] [AT1G53160] [AT5G25560] [AT4G35170] [AT2G33810] [AT2G21840] [AT3G57920] [AT1G69170] [AT5G50570] [AT3G15270] [AT1G22000] [AT1G27370] [AT3G62420] [AT5G50670] [AT4G24270] [AT5G43270]	2.10E-03

Table S5. The expression levels of circRNAs in the miR156a-related network

Tools	Name	Treatment									
		E2LLA	E2HL30A	E2HL30B	E2HL2HA	E2HL2Hb	TNR1LLA	TNR1HL30A	TNR1HL2HA		
Finder	ATH_circ03766	0	0	0	0	0	0	0.0525	0		
Finder	ATH_circ03768	0	0	0	0	0	0	0.0525	0		
Finder	ATH_circ05254	0	0	0	0.0334	0	0	0	0		
UROBORUS	ATH_circ09695	0	0	0	0	0	0	0	0.0147		
UROBORUS	ATH_circ09697	0	0.0298	0	0	0	0	0	0		
UROBORUS	ATH_circ09698	0.0143	0	0	0.0167	0	0	0	0		
UROBORUS	ATH_circ09699	0.0428	0.1192	0.1124	0.3177	0.2040	0.0759	0.0787	0.1761		
UROBORUS	ATH_circ09700	0	0	0	0.0167	0	0	0	0.0147		
UROBORUS	ATH_circ09701	0.0143	0	0	0	0.0170	0.0190	0.0262	0		
		No treatment		4 CEL		37 CEL		200mM NaCl		300mM mannitol	
UROBORUS	ATH_circ09695	0	0	0	0	0	0	0	0	0.0388	0
UROBORUS	ATH_circ09696	0.0511	0	0	0	0	0	0	0	0	0
UROBORUS	ATH_circ09697	0	0	0	0	0.0261	0	0	0	0	0

E2LLA: E2 Low Light bioreplicate A
E2HL30A: E2 High-Light 30 minutes bioreplicate A
E2HL30B: E2 High-Light 30 minutes bioreplicate B
E2HL2HA: E2 High-Light 120 minutes bioreplicate A
E2HL2HB: E2 High-Light 120 minutes bioreplicate B
TNR1LLA: tnr-1 Low Light bioreplicate A
TNR1HL30A: tnr-1 High-Light 30 minutes bioreplicate A
TNR1HL30B: tnr-1 High-Light 120 minutes bioreplicate A
